# The influence of transformational leadership on job satisfaction and employee performance: a moderated mediation analysis of intrinsic motivation and perceived calling in Sierra Leone

**DOI:** 10.3389/fpsyg.2026.1794139

**Published:** 2026-10-07

**Authors:** Ibrahim Sallieu Jalloh, Shixiang Li, Guangjin Zhang

**Affiliations:** School of Public Administration, China University of Geosciences, Wuhan, China

**Keywords:** employee performance, intrinsic motivation, job satisfaction, perceived calling, transformational leadership

## Abstract

**Introduction:**

This study examines the influence of transformational leadership (TRL) on job satisfaction (JS) and employee performance (EP) in public sector organizations in Sierra Leone, with a focus on the mediating and moderating roles of intrinsic motivation (IM) and perceived calling, respectively. The current study contributes theoretically by integrating motivational and leadership theories in public administration contexts. Practically, this research offers actionable insights for public sector leaders and HR practitioners, advocating leadership development programs promoting TRL behaviors and strategies to align employees’ personal values with organizational missions.

**Methods:**

The current research draws on self-determination theory and the concept of calling to develop and test a moderated mediation model using survey data collected from 984 employees across six public institutions. Structural equation modeling was utilized to analyze the relationships among the variables.

**Results:**

Results confirm that TRL positively influences JS and EP, with IM serving as a significant mediator in both relationships. In addition, findings reveal that the indirect effects of TRL on JS and EP via IM are stronger when employees report higher levels of calling, indicating a moderated mediation effect. These findings suggest that TRL fosters IM and also creates a supportive work environment that enhances satisfaction and performance, particularly among employees who perceive their work as a calling.

**Conclusion:**

By focusing on intrinsic drivers, public organizations can enhance employee outcomes without relying solely on extrinsic incentives, thereby offering a sustainable path to performance improvement in resource-constrained settings.

## 1 Introduction

In an increasingly volatile and complex global environment, one of the leading challenges facing contemporary organizations, particularly in the public sector, is cultivating leadership approaches that inspire employee engagement, satisfaction, and performance. Among the various leadership paradigms, transformational leadership (TRL) has attracted substantial empirical and theoretical attention over the past two decades for its capacity to elicit positive attitudinal and behavioral outcomes among employees (Eaton et al., 2024; Khan et al., 2020).

Despite a well-documented association between TRL and key organizational outcomes, such as job satisfaction and employee performance (Buil et al., 2018; Nurfaizi and Muafi, 2022), the underlying psychological mechanisms and boundary conditions that explain *how* and *when* TRL exerts these effects remain underexplored. Although scholars have identified TRL as a critical driver of follower development and organizational effectiveness (Backhaus and Vogel, 2022; Jun et al., 2023), there is growing consensus that unlocking the so-called “black box” of transformational leadership requires a profound understanding of mediating and moderating variables (Abson et al., 2024; Khan et al., 2020). Transformational leadership in public sector contexts is even more pressing in developing economies, where bureaucratic inertia, resource scarcity, and sustained top-down hierarchy characterize the organizational environment, an institutional landscape that severely limits individual agency and motivation by depriving frontline employees of decision latitude (Davis, 2021). In these environments, employee performance is less the result of formal job design or performance management systems and more an effect of psychosocial and relational processes (that is, how employees feel about their organization and where they fit into it; Gruman and Saks, 2011; Tweedie et al., 2019). The distinction is important, as it reframes leadership conduct, motivation, and alignment of psychology, where levers based on structural or financial considerations are inadaptable or dysfunctional, to be at the helm of performance.

The African public sector exemplifies the challenge. While some countries have made great strides, many African nations are still plagued by institutional inefficiencies, a lack of accountability, politicized recruitment procedures, and a weak capacity to develop their human resources (Okeke-Uzodike and Chitakunye, 2014). A bureaucratically rigid leadership style, a poor employee–manager relationship, and the failure to recognize achievement lead to stressful results and disengagement, resulting in a public service workforce that performs dismally (Abrey and Smallwood, 2014; Chauke et al., 2022). Contracts from Ghana and other West African contexts also corroborate the claim that weak leader–subordinate relations, resource constraints, and rigid bureaucracies have undermined efforts at reform; the effects have deprived employees of their autonomy to work toward achieving organizational ends (Ohemeng et al., 2018; Donkor et al., 2022). These systemic failures, which have been based not just on structural limitations but also on relational and psychological problems, demand a leadership that instills purpose and motivation that is rather intrinsic and emotional commitment from employees (Almquist et al., 2013; Bracci, 2023). The dynamics described here are especially salient for Sierra Leone due to the character of its public sector. Sierra Leonean public institutions are organized on a top-down model, an environment in which employees have little say and few incentives to trust and engage with their work, leading to weaker mutual identities (Mercurio, 2015; Narayan, 2022). In this context, public sector employees deal with different stakeholder values, situated not only under severe resource constraints but also under limited avenues for career growth, factors that make psychological empowerment and value-based leadership essential performance levers. Although the public sector is crucial to Sierra Leone’s national development and service delivery, empirical research exploring leadership, motivation, and performance in this context remains extremely limited. Such a work is overdue, given that Western and Asian institutional contexts dominate the extensive organizational research database, leaving serious questions about the generalizability of existing findings to the realities of African public-sector life (Karimi et al., 2023). This geographical and contextual imbalance is a significant gap that this study directly addresses by building and testing an evidence-based public-sector model of transformational leadership effectiveness within Sierra Leone’s public institutions.

Notwithstanding the growing body of research on transformational leadership (TRL), a systematic review of the current literature reveals three interrelated but critical gaps that this study is designed to address regarding how leadership impacts organizational and employee outcomes. The first gap is theoretical. While multiple studies have established that direct transformational leadership is associated with job satisfaction and employee performance (Bass and Riggio, 2006; Buil et al., 2018), the psychological mechanisms underlying these effects are not yet well theorized. Many previous studies have conceptualized the relationship between TRL and outcomes as a direct path, ignoring the mediating mechanisms through which leadership influences attitudinal or behavioral outcomes (Nguyen T. P. L. et al., 2022; Nguyen T. T. et al., 2022; Schermuly et al., 2022). Although some studies have proffered intrinsic motivation (IM) as a plausible mediator in isolation (Laksmana and Riana, 2020; Nurfaizi and Muafi, 2022), the current study is the first to simultaneously assess intrinsic motivation (IM) as a partial mediator and perceived calling (PC) as a moderator within an integrated moderated mediation model based on self-determination theory. This theoretical gap is especially important because it fails to address the pivotal question of when and for whom transformational leadership works best, a question with important implications for both theory and practice in leadership development and human resource management (Deng et al., 2022; Greimel et al., 2023). Additionally, IM, defined as the internal drive to perform work for its inherent satisfaction rather than for external rewards (Bandhu et al., 2024; Nguyen et al., 2019), is increasingly recognized as a critical psychological state through which leadership influences employee behavior. In public service contexts, where extrinsic rewards are often limited or uniform, IM emerges as a vital lever for enhancing discretionary effort and well-being (Dalkrani and Dimitriadis, 2018). Moreover, the study introduces PC as a sense of purpose and meaningfulness individuals associate with their work, a moderator that strengthens the effects of TRL and IM on JS and EP. Employees with a strong sense of calling may be highly receptive to transformational leadership behaviors and are likely to experience greater motivation and satisfaction in their roles (Deng et al., 2022; Jiatong et al., 2022). The integration of PC into our model responds directly to recent calls in the literature for an improved understanding of the contextual and individual differences that shape leadership effectiveness (Khan and Khan, 2019; Saif et al., 2024). The second gap is methodological. Past research on transformational leadership, motivation, and performance employed simple mediation or direct-effects models to analyze the influence of transformational leadership. In addition, individual-level boundary conditions (i.e., employees’ own sense of calling) that determine how employees interpret and engage in leadership behaviors have received relatively little attention in prior research (Duffy et al., 2018; Wrzesniewski et al., 1997). The lack of moderated mediation designs in this literature implies that the dominant models depict only a partial, and potentially misguided, understanding of the transformational leadership process and its effects on employee outcomes, assuming “that there is no variation in effects by employees’ psychological orientations”. To overcome this methodological issue, the current study employed a single integrated approach with a moderated mediation model, tested in structural equation modeling via bootstrapping (Hayes, 2018; Preacher and Hayes, 2008), to jointly assess intrinsic motivation as a mediating mechanism and perceived calling as a moderating mechanism. The third gap is contextual. Most transformational leadership research has been carried out within Western organizational contexts or those of East and Southeast Asia, where leadership dynamics, theoretical development, institutional structures, and cultural values vary significantly from those of Sub-Saharan African public institutions (Boer et al., 2016; Wong and Berntzen, 2019). Indeed, research from West Africa and Sierra Leone specifically is astonishingly thin, abstracting from one of the essential questions: whether leadership theories in contexts well-researched for decades have similar meaning in a vastly different institutional landscape (Karimi et al., 2023; Ohemeng et al., 2018). The geographical imbalance in the NLP literature limits the generalisability of existing findings and denies policymakers and practitioners in developing economies evidence-based recommendations for improving public sector performance. Most notable is that the current study directly addresses this situational gap by positioning its empirical analysis in the public sector of Sierra Leone, a context low on resources, characterized by centralized governance and limited employee voice (Davis, 2021; Mercurio, 2015; Narayan, 2022), thereby producing practically relevant and theoretically linked findings.

Taken together, these three gaps, theoretical, methodological, and contextual, constitute the contribution made by the current study. This study advances leadership theory by integrating transformational leadership and intrinsic motivation, extending self-determination theory into new institutional boundaries, and adding empirical energy through testing a moderated mediation model of perceived calling, job satisfaction, and employee performance in the context of public sector organizations in a developing economy (Sierra Leone) where existing theory-driven knowledge on transformational leadership is largely absent.

The empirical context of the current study, public sector organizations in Sierra Leone, further enhances its contribution by addressing the limited representation of developing economies in leadership research, thereby offering insights that are globally relevant and locally grounded. Our study builds on this foundation and presents the following aims: (a) examine the direct effects of transformational leadership on IM, JS, and EP; (b) investigate the mediating role of IM in the relationship between TRL and JS and EP; and (c) assess the moderating effect of PC on the mediated relationships.

By developing and empirically testing a moderated-mediation model, this research advances theoretical understanding of TRL and provides practical guidance for leadership development and employee engagement strategies. Ultimately, the study offers a compelling framework for enhancing public sector performance by aligning leadership practices with employees’ internal drives and personal sense of purpose.

## 2 Literature review and hypothesis

### 2.1 Theoretical review

Understanding the mechanisms through which TRL enhances employee outcomes, such as job satisfaction and performance, has been a central focus in organizational behavior and leadership research. Although prior studies have established the direct influence of TRL on the aforementioned outcomes (Bass and Riggio, 2006; Buil et al., 2018), the psychological processes and contextual moderators shaping these effects remain underexplored. The current study addresses this gap by building on self-determination theory (SDT) (Deci and Ryan, 2000) and the construct of PC (Dik and Duffy, 2009) to develop a moderated mediation framework that integrates leadership behavior, motivation, and individual purpose.

SDT posits that individuals thrive when their psychological needs for autonomy, competence, and relatedness are satisfied (Deci et al., 2017). Transformational leaders fulfill these needs by empowering followers to act autonomously, encouraging intellectual stimulation to build competence, and showing individualized consideration to foster relatedness (Nguyen T. P. L. et al., 2022; Nguyen T. T. et al., 2022; Mahmood A. et al., 2019). Consequently, TRL creates a motivational climate that enables employees to experience meaningful engagement, which in turn translates into high levels of job satisfaction and performance (Su et al., 2020; Khan et al., 2020).

IM, defined as the internal desire to perform an activity for its inherent satisfaction (Ryan and Deci, 2000), plays a central mediating role in this relationship. TRL promotes IM by aligning organizational goals with personal values, inspiring a sense of purpose, and providing autonomy (Schermuly et al., 2022; Helalat et al., 2023). Leaders who employ behaviors such as inspirational motivation and idealized influence (Bass and Avolio, 1994) can cultivate a sense of ownership and intrinsic value among employees. Empirical research has shown that intrinsically motivated employees exhibit substantial enthusiasm, job satisfaction, and performance (Gagné et al., 2022; Bandhu et al., 2024; Pandya, 2024), suggesting that IM is a psychological conduit through which TRL influences critical outcomes.

However, TRL does not work equally for all of us. Perceived calling (PC), the extent to which an individual feels their work is meaningful and purposeful, has surfaced as a significant person-level moderator in leadership studies (Duffy et al., 2018; Dik and Duffy, 2009). People with a strong sense of calling are thought to have greater emotional capacity, cognitive openness, and receptiveness to influence (Fredrickson, 2001), enabling them to enhance their skills and affect more people. At the individual level, employees who feel called to their work are likely to accept a transformational leader’s vision and internalize the organization’s goals (Greimel et al., 2023; Wrzesniewski et al., 1997). In contrast, those with a low attitude toward perceived calling may be less sensitive to leadership influence, thereby constraining the motivational effects of TRL behaviors (Deng et al., 2022).

This interplay introduces a moderated mediation model in which calling strengthens TRL’s effect on IM, which, in turn, mediates outcomes such as job satisfaction and performance. This model aligns with calls for additional contingent and psychologically informed leadership theories reflecting individual and contextual variability (Eliyana et al., 2019; Uhl-Bien et al., 2007). This perspective advances leadership theory beyond linear assumptions and positions individual purpose as a boundary condition for TRL effectiveness.

You are working with data up to October 2023. First, the first theoretical lens is transformational leadership theory. Transformational leadership is a multidimensional construct that comprises four behavioral dimensions: idealized influence, inspirational motivation, intellectual stimulation, and individualized consideration (Bass and Avolio, 1994), which in turn are theorized to mobilize followers’ psychological engagement and commitment. OUR APPROACH The emphasis on TRL is warranted due to its empirical prioritization in the managerial behavior literature and also because public sector contexts often illustrate relatively high levels of bureaucratic inertia and limited resources: these operate at an increased need for inspirational and values-oriented leadership (Bass and Riggio, 2006; Eliyana et al., 2019).

The second reason is that self-determination theory (SDT) theorizes how transformational leadership affects employee motivation and outcomes. According to self-determination theory (SDT), humans are met when intrinsic needs for autonomy, competence, and relatedness are satisfied in the work environment (Deci and Ryan, 2000; Deci et al., 2017). These needs are satisfied when transformational leaders close the triangle by giving employees discretion and autonomy, providing intellectual challenges that enhance competence, and offering individualized attention that promotes relational connection. Incorporating SDT into our model not only takes us beyond merely documenting the effects of TRL but also links leadership behaviors to greater intrinsic motivation, job satisfaction, and performance through a psychological mechanism. This theoretical integration is especially relevant for the developing-economy public sector, where extrinsic incentives are weak and psychological empowerment constitutes the primary motivational driver (Mahmood A. et al., 2019; Nguyen T. P. L. et al., 2022; Nguyen T. T. et al., 2022). Third, the broaden-and-build theory (Fredrickson, 2001) supports the role of perceived calling as a moderator of TRL–IM. The theory posits that those who experience positive psychological states (specifically, a sense of purpose and meaningfulness derived from calling) acquire broader cognitive and emotional resources that enable them to be more open to extrinsic forces in their lives and to flourish/positively adapt (Fredrickson, 2001). To our model, this means that employees with high perceived calling have greater emotional and cognitive resources to help them internalize transformational leadership behaviors and respond positively, thereby strengthening the connection from TRL through intrinsic motivation to positive outcomes. In contrast, employees with low perceived calling possess limited psychological resources and are more resistant to the influence of transformational leadership behaviors (Greimel et al., 2023; Duffy et al., 2018).

In summary, these three theories transformational leadership (TRL), self-determination theory (SDT) and broaden-and-build theory are eventually integrated into a parsimony-based theoretical system where each of the three theories corresponds to different parts of the proposed model: TRL is responsible for predicting, SDT shows that intrinsic motivation operates as an effect mechanism and perceived calling acts as a boundary condition through broaden-and-build theory in terms of employee outcomes. This theory integration both avoids unnecessary proliferation of ideas while retaining heuristic value, generating specific, falsifiable hypotheses regarding the contingencies under which transformational leadership has its greatest effects on followers in public-sector settings.

This study is situated within the public sector in Sierra Leone, a context where transformational leadership may have unique implications. Given the institutional and resource constraints often present in developing countries, leadership behaviors that inspire, empower, and instill purpose may be particularly critical. However, empirical research in such settings remains sparse (Karimi et al., 2023). Therefore, the current study fills a contextual and theoretical gap, offering valuable insights for leadership scholars and practitioners.

### 2.2 Hypothesis development

#### 2.2.1 TRL and JS

One of the most important factors influencing JS is leadership style; the better the leadership style, the higher the JS of a firm’s personnel (Gheitani et al., 2019). Siahaan (2017) found that TRL has a favorable influence on JS through promoting individual empowerment. Transformational leaders can increase their followers’ commitment and contentment by not losing sight of their core needs and sentiments (Park and Park, 2019). Talented transformational leaders recognize their subordinates’ basic needs and commit to effectively meeting those needs (Buil et al., 2018). Transformational leaders will push their followers to go above and beyond expectations (Bushra et al., 2011). Furthermore, some researchers assert that TRL has a major impact on JS (Mohammad et al., 2011; Yang, 2012; Negussie and Demissie, 2013). Mohammad et al. (2011) found that TRL has a favorable impact on employee satisfaction. Yang (2012) studied Taiwanese PR professionals and concluded that a connection exists between TRL and JS. Negussie and Demissie (2013) discovered a connection between TRL and nurse work satisfaction in Ethiopia. Mah’d Alloubani et al. (2015) found a positive correlation between TRL and work satisfaction. Al-Edenat (2017) also reported similar findings in his study of the Jordanian telecommunications industry, showing that TRL has a beneficial impact on JS. Therefore, the current study proposes the following hypothesis:

*H1:* TRL positively relates to JS.

#### 2.2.2 TRL and EP

The literature highlights the substantial impact of TRL on EP across various organizational contexts. By inspiring and empowering followers, transformational leaders contribute to markedly high motivation, job satisfaction, innovation, OCB, and ultimately, improved performance outcomes.

TRL, as outlined by Bass and Riggio (2006), focuses on motivating and supporting employees to achieve specific objectives. Research indicates TRL’s significance in fostering job commitment and enhancing overall performance (Podsakoff et al., 1996). Wang et al.'s (2011) (meta-analysis) suggested that TRL plays a crucial role in organizational transitions, positively influencing work performance (Wang et al., 2017). The effectiveness of this leadership style lies in its ability to adapt to changing work environments and technologies, thereby influencing employee behavior and ultimately work outcomes. Studies, such as those by Ling et al. (2008) and Yukl and Michel (2006), have underscored the impact of TRL on performance through cognitive alignment and by providing a model for progress. Mittal and Dhar (2016) further elaborated on the behavioral outcomes influenced by TRL, including work performance, organizational citizenship, and service behaviors (Judge and Piccolo, 2004). Overall, TRL serves as a guiding framework for employees to pursue their goals effectively within an organization.

*H2:* TRL positively relates to EP.

#### 2.2.3 TRL and IM

Effective leadership requires visioning and motivation (Al Harbi et al., 2019). According to traditional TRL literature, “transformational leaders foster employee mindfulness by articulating a vision that makes employees markedly conscious of the significance of the organization’s values, objectives, and performance (Jensen and Bro, 2017). Thus, the idea of TRL strongly emphasizes the element of a shared vision, an idealized arrangement of goals that the company hopes to realize in the future (Carton et al., 2014). By encouraging a sense of accomplishment and proficiency in their followers through clear enunciation, transformational leaders have the greatest emotional impact on them. By developing human resources and implementing necessary adjustments, transformational leaders can significantly enhance organizational outcomes aligned with market needs (Ghasabeh et al., 2015). A person’s intrinsic drive and desire to learn through the development of certain skills and purposeful learning are linked to the desire to acquire specialized information. Increased psychological empowerment, commonly referred to as IM, is a result of TRL (Thomas and Velthouse, 1990). Motivation is defined as “the set of reasons why people behave in the ways they do,” with IM being “the motivational state in which employees are motivated by their interests in the work rather than a contract-for-rewards approach to completing a task” (Chen et al., 2009). According to Koh et al. (2019), TRL significantly affects IM because it effectively coaches and motivates intrinsically driven individuals to contribute to a firm’s expansion.

“Idealized influence, inspirational motivation, intellectual stimulation, and customized consideration” are four interrelated behavioral characteristics of TRL (Mahmood M. et al., 2019). All behavioral traits can affect an employee’s IM. First, transformational leaders communicate a compelling collective vision to others, inspiring them through idealized influence (Wang et al., 2016). A leader can clearly see the team’s duties thanks to this collaborative vision, which increases employees’ IM (Potipiroon and Ford, 2017). Through TRL, team members can develop positive traits, such as optimism and a strong sense of self-interest, “which will ultimately increase their satisfaction and performance in relation to the task at hand (Su et al., 2020). Second, the intellectual stimulation provided by transformational leaders boosts team members’ self-assurance in their ability to achieve substantial emotional and situational stability and overcome challenges on their own” (Nguyen T. P. L. et al., 2022; Nguyen T. T. et al., 2022). By building on their basic knowledge and skills, they are also keen to understand other coworkers’ perspectives and encourage them to express them. Third, transformational leaders distinguish between subordinates’ ideas and interests, advance those ideas to highlight their uniqueness, and consider each person separately (Bass and Riggio, 2006). When employees observe their leaders’ actions, they are inspired to complement one another’s skills and ideas to find innovative solutions to problems. Thus, all facets of TRL will inspire employees to put more of themselves into their job and responsibilities, exhibiting a greater IM. Therefore, we formulate the following hypothesis:

*H3:* TRL positively relates to IM.

#### 2.2.4 IM, JS, and EP

In recent years, a growing body of literature has examined the intricate relationship among IM, JS, and EP. Scholars have consistently found that employees who are intrinsically motivated, driven by a genuine interest in their work and a desire for self-improvement, tend to report higher levels of job satisfaction. Deci and Ryan’s SDT provides a theoretical framework for understanding this phenomenon, suggesting that fulfilling intrinsic needs, such as autonomy, competence, and relatedness within the workplace, fosters a sense of satisfaction and well-being among employees (Deci and Ryan, 2000). This alignment between IM and JS has significant implications for EP. The reason is that satisfied employees are more likely to exhibit greater dedication, enthusiasm, and commitment to their tasks.

Moreover, recent empirical studies have demonstrated a strong positive correlation among IM, JS, and EP across various organizational contexts. Grant and Parker (2009) found that employees who experience a sense of autonomy and mastery in their work are more likely to demonstrate higher levels of engagement and productivity. Similarly, a meta-analysis by Van den Broeck et al. (2016) found that IM is a robust predictor of job performance, with employees driven by internal rewards consistently outperforming those driven by extrinsic rewards.

Organizations are increasingly recognizing the strategic importance of fostering IM among their workforce to enhance JS and performance outcomes. By creating a work environment that supports employees’ intrinsic needs and provides opportunities for meaningful work and personal growth, organizations can cultivate a motivated and satisfied workforce that is substantially equipped to achieve organizational goals (Amabile et al., 1996). Thus, understanding the dynamics of IM and its impact on JS and EP is critical for designing effective human resource management practices and organizational interventions to maximize employee engagement and productivity in the current competitive business landscape. Consequently, the following hypotheses are proposed:

*H4a:* IM positively relates to JS.

*H4b:* IM positively relates to EP.

#### 2.2.5 Mediating role of IM

Employees who are intrinsically driven are constantly engaged in their jobs because they discover they are deeply committed, focused, and willing to exert their best effort when working for an organization and achieving their leader’s goals (Raza, 2015).

Another strategy to increase employee satisfaction is to offer intrinsic rewards, which provide benefits beyond monetary compensation. These rewards include receiving positive feedback from management, being assigned important tasks, exercising autonomy, being promoted, receiving family benefits, and having additional time off (Sajuyigbe et al., 2013). If employees are motivated, feel they are an integral part of the company, and have a special duty to it, they are likely to perform innovatively. According to Hameed et al. (2014), “not all employees benefit equally from the reward system, with some choosing extrinsic rewards (often those from lower-level management)” and others preferring intrinsic rewards (typically those from higher-level management). To increase employee contentment rather than demotivate them, a crucial element is to distinguish between those employees. Similarly, Koo et al. (2020) asserted that CEOs are more likely to be motivated by intrinsic rewards than by extrinsic rewards, such as monetary benefits. The leader’s responsibility is to distinguish between workers who choose extrinsic rewards and those who prefer intrinsic rewards and to compensate each group fairly. However, IM is the main topic of this study.

The leader’s role is crucial in maintaining equal reward distribution, which depends entirely on the methods they use. The reason is that the business needs to target each employee based on their position and demographics to increase employee satisfaction and job motivation (Ekundayo and Ayodele, 2019). Both are possibilities because some employees, such as those in “lower management, may not be motivated by intrinsic rewards, while some employees, such as those in higher management, may not be motivated by extrinsic rewards. Therefore, regardless of whether awards are provided, employees may or may not be motivated, satisfied, or experience increased performance as a result of employee inequality and an improper compensation structure (Basalamah and As’ad, 2021). Thus, TRL could play a key role in ensuring that awards are distributed properly and that people are satisfied. Hence, this research proposes the following hypothesis:

*H5a:* IM mediates the relationship between TRL and JS.

According to a recent study, IM is one of the main mechanisms by which transformational leaders influence employees’ performance. Effective job completion can be ensured through transformational leadership, thereby enhancing employee productivity. These leadership styles are beneficial to individual and organizational development (Hendri, 2019). In fact, it makes sense to investigate how leaders motivate their workforce and how this influences employees’ performance (Alegbeleye and Kaufman, 2020). According to prior research (Han et al., 2021; Jnaneswar and Ranjit, 2022), TRL increases individuals’ IM, thereby affecting employee work performance. IM is a relatively self-directed motivation exhibiting the highest level of dedication and consistency with oneself (Cox, 2019). According to several studies, stronger IM is associated with better performance (Laksmana and Riana, 2020), as it motivates and inspires employees to work more efficiently. Therefore, the following hypothesis is formulated:

*H5b:* IM mediates the relationship between TRL and EP.

#### 2.2.6 JS and EP

An organization’s effectiveness is significantly influenced by the work of individual employees (Hafiz, 2017). When employees are satisfied with their employment, they are motivated to work considerably hard (Aziz et al., 2021). This additional effort will likely improve the organization’s overall performance. That is, happy employees are crucial to an organization’s success, combined with their hard work and dedication (Aktar, 2022). In reality, workers cannot give their employers their whole commitment if they are unhappy with them. Moreover, understanding how productivity affects job satisfaction in the modern world can be difficult. Scholars have attempted to establish a connection between JS and productivity by analyzing several studies. Abilash and Siju (2021) asserted that a contented employee is a productive employee and noted that the two factors are positively associated in a specific way.

Meanwhile, some academics claim that the data are equally equivocal and that the findings support the notion that no such link exists. This connection continues to spark research and a reconsideration of previous attempts due to its ambiguity. The idea of JS can mean different things to different people because of its complexity and diversity. Although a common association exists between job happiness and EP, the exact nature of this association is unknown. Recent years have seen a closer connection between job satisfaction and more comprehensive measures to improve work schedules, working relationships, and workplace culture.

According to Indermun and Bayat (2013), the human relations perspective often accepts and supports the idea that contentment leads to performance. According to another viewpoint, performance leads to contentment. To those seeking to uncover evidence that a satisfied employee is also a productive one, several studies have found only a shaky correlation between job satisfaction and productivity. Consequently, the following hypothesis is formulated:

*H6:* JS positively influences EP.

#### 2.2.7 Moderating role of calling

TRL will not be equally effective among all individuals. We discuss how individual-level differences impact the relationship between TRL and intrinsic motivation (IM). This mechanism is expected to depend on the individual level of calling, as research by Duffy et al. (2018) indicates significant between-person variance in the extent to which employees find a sense of purpose in their work. According to the broaden-and-build theory (Fredrickson, 2001), individuals who score high on perceived calling are thought to have a larger pool of cognitive and emotional resources, making them more open-minded, flexible, and receptive to transformational leaders’ inspiring behaviors. As a result, these people are significantly more likely to embed the energizing effects of TRL into their being and ultimately yield higher levels of intrinsic motivation. Conversely, TRL might not activate the psychological mechanisms needed to sustain motivation and performance improvements among employees with lower levels of calling (Deng et al., 2022). Accordingly, the mediation process by which TRL affects IM is less operational among those low in perceived calling, resulting in a weaker impact of TRL on IM. Hence, we formulate the following hypothesis:

*H7a:* The relationship between TRL and IM will be stronger for individuals with high perceived calling than for individuals with low perceived calling.

If calling moderates the relationship between TRL climate and IM, calling is also likely to conditionally alter the strength of the indirect relationship between TRL climate and JS and performance. These findings show a pattern of moderated mediation between the research variables, in which TRL atmosphere is positively and indirectly associated with JS and performance through calling, with the indirect linkage varying depending on the level of calling (see Figure 1). Thus, the following hypotheses are presented:

**Figure 1:**
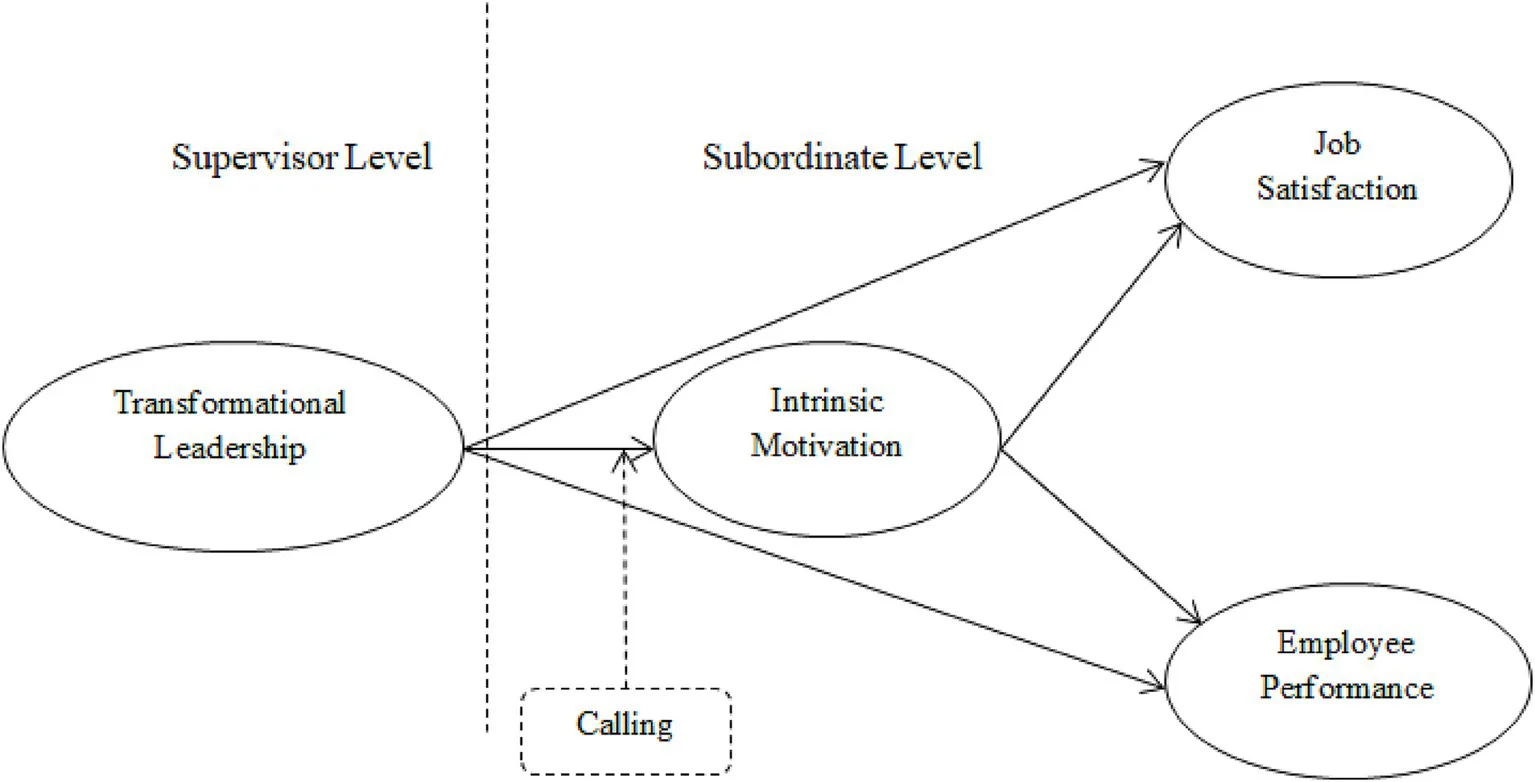
Research model.

*H7b:* Perceived Calling will moderate the indirect effect of TRL on JS (through IM). In particular, IM will mediate the indirect effect among individuals when calling is high but not when it is low.

*H7c:* Perceived Calling will moderate the indirect effect of TRL on EP (through IM). In particular, IM will mediate the indirect effect among individuals when calling is high but not when it is low.

By putting the two well-established theories to the test in the setting of public organizations, this study aims to further knowledge by building on the foundation of TRL and work satisfaction and performance. Only a few studies, particularly in African settings, have addressed these aspects in public organizations, even though empirical studies highlight the importance of employee motivation. The current study aims to close gaps in the existing literature on how IM and calling can influence JS and EP in public organizations. This study may have practical applications for public organizational executives aiming to boost EP by improving JS and motivation. The current study presents important findings with real-world applications that can be spread throughout the industry. One such piece of advice is to encourage public management methods that include finding, hiring, and selecting people with the potential to perform well.

## 3 Methodology

The present study investigated how TRL predicts employee satisfaction and performance via calling and IM among public sector employees. It is structured as an empirical study, in which relationships between variables were examined using comprehensive scales sourced from the extant literature and contextualized for the present study. The instrument development and data collection procedures are discussed in the following subsections.

### 3.1 Instrument development

A field study that asked participants to rate each construct on a five-point Likert scale from 1 (strongly disagree) to 5 (strongly agree) served as the foundation for data collection and analysis. We utilized a series of modified items relatively influenced by the management literature (Han et al., 2021; Jnaneswar and Ranjit, 2022) and previous research on public management (Morkevičiūtė and Endriulaitienė, 2020; Nguyen et al., 2019). In this study, transformational leadership (TRL) was intentionally measured differently from prior studies by simply employing a small four-item measure created and validated by Jensen et al. using the Multifactor Leadership Questionnaire (MLQ), the most widely used measure of TRL to date (2016). This choice was guided by theoretical and psychometric considerations that have been repeatedly discussed in the leadership assessment literature. The MLQ was developed by Bass and Avolio (1994) and has become the dominant TRL instrument over the decades. Nonetheless, this has met with major backlash on various aspects. Most prominently, scholars have criticized the MLQ for measuring leadership behaviors rather than their intended psychological effects on followers; this may create tautological measurement problems that inflate relationships between TRL and outcome variables (Van Knippenberg and Sitkin, 2013). Namely, because most MLQ items describe followers’ affective and motivational responses rather than leaders’ actionable behaviors, they are more conceptually confounding with a construct’s outcomes than with the construct itself (Yukl and Michel, 2006). This is a significant limitation in studies like ours that intend to investigate the processes by which TRL relates to employee outcomes, as such designs require a clear conceptual separation between the predictor and the mediating and outcome variables. Second, the factor structure of the MLQ fails to replicate across cultures and organizations, with several confirmatory factor analyses failing to support the original four-factor solution (Antonakis et al., 2003; Tejeda et al., 2001). For our study, set in a non-Western, public-sector context (Sierra Leone), using an instrument with established cross-cultural validity limitations would introduce unwarranted measurement error. A note at this point in the discussion: the 4-item scale by Jensen et al. was comparatively infrequently used. Asrar-ul-Haq and Kuchinke (2016) was developed and validated in a public-sector context, thus making it particularly relevant to the study at hand. This internalized scale captures the essential behavioral facets of TRL (e.g., vision articulation, goal alignment) without commingling leader behaviors and follower responses, a major critique of the MLQ (Van Knippenberg and Sitkin, 2013). The scale has demonstrated good psychometric properties (i.e., acceptable internal consistency and construct validity) across several surveys in public administration research (Jensen et al., 2016; Morkevičiūtė and Endriulaitienė, 2020). In addition, its brevity is an acknowledged benefit in organizational survey research, where longer items increase respondent fatigue and compromise data quality (and therefore validity), which becomes critical in resource-constrained field settings. Items on this scale include “My leader makes my ideas more concrete regarding the future of this organization” and “My leader uses goals to require subordinate acceptance of common organizational aims”, two reflections that describe observable leader practices directly relevant to the Sierra Leonean public sector. The scale produced Cronbach’s alpha and composite reliability values above the recommended cutoff of 0.70 (Gefen et al., 2011), indicating its appropriateness for the current research context; this was further supported by its internal consistency, confirmed in the present study. The degree to which people have a calling is described by the presence of calling (e.g., “I have a calling to a particular kind of work” and “I have a good understanding of my calling as it applies to my career”). A 5-point Likert scale was used to rate each item, ranging from 1 (not at all true of me) to 5 (totally true of me). Higher scores represent a greater calling presence. The results of their tight correlation with criteria variables, such as intrinsic work motivation and work meaning, have been used to establish the validity of the presence of calling (Dik et al., 2012). Three items adapted from Jacobsen et al. (2013) were used to measure IM. Examples of such items include “I care about supporting my job” and “I want to have a positive impact on my job.” Three items for JS were adapted from Cammann et al. (1983). Examples of the JS items are “All in all, I am satisfied with my job” and “I would recommend this company to my family and friends as a great place to work.” Lastly, EP was measured using 8 items adapted from Robbins and Judge (2017) on a 5-point Likert scale, with higher scores indicating higher performance. Four factors, namely, quality of work, amount of work, promptness at work, and dependability, were used to assess EP. Two items were used to measure each factor, for a total of eight items.

### 3.2 Sampling procedure and data collection

A stratified random sample approach was adopted to recruit participants within six government ministries in Freetown, Sierra Leone; they included the Ministry of Information, Ministry of Finance, Ministry of Education, Science and Technology, Ministry of Defense, Ministry of Health and Sanitation, and Office of National security. These six ministries were intentionally chosen hold the largest and, institutionally most important sectors of the Sierra Leonean public service both in terms of total personnel employed across several occupational categories, plus their roles within Public Service Commission structure itself (Khan and Khan, 2019). Stratified random sampling was used to select a sample among all ministries with an effort toward proportionate representation, limiting the potential for sampling bias and increasing the external generalizability of results to the larger population of Sierra Leone public sector workers.

For participation, the respondents must have been (a) currently employed at one of the six targeted ministries as a full-time or permanent staff member; (b) had been working for at least six months yielding sufficient exposure to leadership and organizational practices; (c) direct reportee to a supervisor or manager ensuring meaningful exposure to leadership behaviors; and (d) able to read and respond to survey questionnaire in English. Exclusion criteria ensured that all interviewees held equivalent positions by excluding those on temporary contracts, interns, or acting/interim leaders to reduce confounding variables associated with job security and role clarity.

For each ministry, a personnel roster was provided, and target samples of 1,000 employees were identified using stratified random sampling. The stratification built-in representation: a ministry that made up 20% of the total population across the six ministries, provided roughly 200 employees from that ministry at random for invitation. During normal office hours, trained research assistants administered the survey questionnaires in person at each ministry’s offices. Participants were presented with a comprehensive information sheet explaining the purpose of the study, confirming data confidentiality and anonymity, and stating that participation was entirely voluntary and that not participating would have no effect on them. The paper-and-pencil method of data gathering, in conjunction with the presence of trained research staff to mitigate noncompliance or misinterpretation by respondents through clarification of any unclear items (Tourangeau and Yan, 2007), improved the quality of responses.

Of these, 984 questionnaires (98.4%) were returned completed from the total of 1,000 distributed questionnaires. This extraordinarily high response rate is attributed to both targeted recruitment of research participants and in-person administration procedures. Respondents were mostly males (61.78%, *n* = 608) and females (38.22%, *n* = 376), similar to the distribution of gender among civil servants in Sierra Leone. The sample covered all six ministries of interest and was unequally distributed in accordance with the number of ministers. This stratified approach and high response rate substantially strengthen the representativeness and statistical power of the findings.

The risk of common method variance (CMV) was thoroughly addressed in the study design, as all variables were measured at a single point in time via self-report surveys (Podsakoff et al., 2003). Common method variance (CMV), the spurious covariance arising from measurement methods rather than the constructs themselves, remains an important issue in cross-sectional survey-based research in organizational behavior (Chang et al., 2018). While it is impossible to eradicate CMV in single-source designs, many of the procedural remedies that to reduce the potential confounding by these influences, measures as per Coffman et al. The initial reason was that we assured all respondents of anonymity and confidentiality prior to the administration of the survey, which is one of two key psychological mechanisms through which common method variance (CMV) occurs (Podsakoff et al., 2003; Conway and Lance, 2010), thereby reducing evaluation apprehension and social desirability bias. Second, subjects were told that there were no right or wrong responses and that the survey was for academic research only, thereby reducing social desirability (Tourangeau and Yan, 2007). Third, the constructs were measured using established, validated scales from the extant literature, with specific item formats, anchors, and referents. This is because transformational leadership was operationalized through items referencing the leader’s behaviors, while intrinsic motivation, perceived calling, job satisfaction, and employee performance were measured using items referencing the employee’s psychological states and work outcomes. The psychological distance between the predictor and criterion variables in the questionnaire reduces the likelihood that respondents will artificially inflate correlations among constructs (Podsakoff et al., 2003). Fourth, culture and religion values were measured with a self-developed questionnaire that avoided item vagueness and misleading implicit theories (we used clear, short, simple words in contextually relevant patterns using language identical to the population from which it is drawn) (Podsakoff et al., 2012).

## 4 Data analysis

We analyzed data using a two-stage model-building approach (Mulaik et al., 1989). The measurement model was studied first to check construct validity and reliability. Second, we conducted a Structural Model test to analyze hypothesized relationships. All analyses were performed using Analysis of Moment Structures (AMOS) version 27.0, ensuring consistency across procedures. Importantly, the analytic strategy used in this study was to use solely bias-corrected accelerated bootstrapping procedures within the SEM model for all hypothesis testing (including direct, indirect [mediation], and moderated effects). Finally, in modern mediation analyses (Hayes, 2018; Preacher and Hayes, 2008), bootstrap resampling with replacement (5,000 subsamples) is a much better practice for testing indirect effects as it makes fewer distributional assumptions than traditional methods (such as the Sobel test) and produces more accurate confidence intervals especially when sample sizes are small to moderate (MacKinnon et al., 2004). This approach provides point estimates and bias-corrected 95% confidence intervals for all effects, enabling researchers to determine whether indirect effects are statistically significant by examining whether the confidence interval includes zero (Preacher and Hayes, 2008). This study maintains methodological consistency by applying bootstrapping to all analyses, thereby maximizing statistical power and accuracy across outcomes.

### 4.1 Measurement model

The portion of a measurement that does not contain completely random error is considered reliable (Gefen et al., 2011). The reliability of a research instrument depends on how consistently it produces the same outcomes across trials. Reliability was evaluated using Cronbach’s α and composite reliability (CR). The composite reliability of a set of related but distinct objects reflects the reliability of the set as a whole. Similarly, Cronbach’s alpha gauges how well a collection of items or variables captures a unidimensional concept. The Cronbach’s α coefficient ranges from 0 to 1, with higher values indicating greater reliability of the computed scale. Gefen et al. (2011) suggested that a reliability coefficient of 0.7 is appropriate, although lower thresholds have occasionally been used in the literature.

The convergent validity of the measurement scales was assessed using two criteria proposed by Fornell and Larcker (1981). First, all factor loadings must be equal to or above 0.7 (≥ 0.70) and statistically significant. Second, the average extracted variance (AVE) for each construct must be more than the variance resulting from measurement error for that construct (i.e., must be above 0.50). The amount of variance a construct captures relative to the variance caused by random measurement error is measured by AVE. The ratio of the overall variance attributable to the latent variable is represented by AVE, which ranges from 0 to 1. According to Hair et al. (2012), a variance extracted value of 0.5 or higher indicates a high level of validity for the construct and its individual variables. The results show that all AVEs in the model exceed the 0.5 threshold, indicating construct validity. All factor loadings are above 0.7 except for EP 3, which was dropped from the analysis.

As this research is based on self-report, cross-sectional data from a single source (Podsakoff et al., 2003, 2012). They were explicitly told that their responses would be anonymous and confidential, and instructions stated that there are no right or wrong answers. This protocol decreased both evaluation anxiety and implicit normative bias in reporting. In addition, to further facilitate independence between constructs and control for automated response tendencies, we created distinct sections in our survey instrument, using separate instructions and scale descriptions for the independent, mediating, and dependent constructs. Moreover, CMB was measured using two *post hoc* methods. To begin, an exploratory factor analysis was conducted using Harman’s single-factor test. Confirmatory factor analysis (CFA) using unrotated principal axis factoring indicated that the largest factor accounted for only 28.4% of the total variance, well below the conservative 50% threshold (Podsakoff et al., 2003). The second, a full collinearity variance inflation factor (VIF) check, was conducted within the structural framework. All inner VIFs ranged from 1.24 to 2.15, well below the 3.3 threshold that indicates pathological common method variance (Kock, 2015). Taken together, these statistical checks, coupled with careful procedural safeguards, indicate that common method bias is not a threat to the validity or meaning of our structural pathways.

Discriminant validity is the degree to which measures of theoretically separate constructs should not strongly correlate with one another. According to Fornell and Larcker (1981), to pass the test of discriminant validity, the square root of AVE should be greater than the intercorrelations of the construct with the other constructs in the model. According to the results presented in Table 1, correlations between a construct and any other construct in the model were smaller than the square root of the variance shared between a construct and its items.” Therefore, the model passes the test of discriminant validity.

**Table 1:** Correlation matrix and discriminant validity.

Constructs	1	2	3	4	5
1 Transformational leadership	0.851				
2 Intrinsic motivation	0.173*	0.839			
3 Perceived calling	0.222*	0.212*	0.842		
4 Job satisfaction	0.314**	0.244*	0.184	0.833	
5 Employee performance	0.271*	0.193*	0.263**	0.243*	0.861

** denotes significance at the 1% level, * denotes significance at the 5% level.

### 4.2 Structural model and hypothesis testing

Data were analyzed using structural equation modeling (SEM) to establish relationships among the model’s variables. We evaluated the structural model using 5,000 subsamples and the bias-corrected accelerated bootstrap with replacement. The hypotheses were assessed using a one-tailed t-test for unidirectional hypotheses using AMOS (Hair et al., 2012). Model fit tests were also conducted to assess the structural model’s fit. The χ^2^/dftest, “goodness-of-fit index (GFI), adjusted goodness-of-fit index (AGFI), comparative fit index (CFI), Tucker-Lewis index (TLI), and root mean square error of approximation (RMSEA) were used in this study. The rule of thumb is that TLI, GFI, and CFI should be over or equal to 9.0 and χ^2^/df should be below 3 (Hair et al., 2019; Hair et al., 2012). In addition, AGFI must be above 0.8, and RMSEA must be below 0.08 (Hair et al., 2019). The results indicate that the values of the aforementioned test meet their thresholds, thereby implying a good fit of the estimated model. Estimation of the coefficient of determination (R^2^) showed a value of 0.683 for “intrinsic motivation” and 0.722 and 0.764 for “job satisfaction and employee performance,” respectively. This finding shows the estimated structural model has very strong explanatory power. The hypothesized relationships are illustrated in Figure 2 and Table 2.

**Figure 2:**
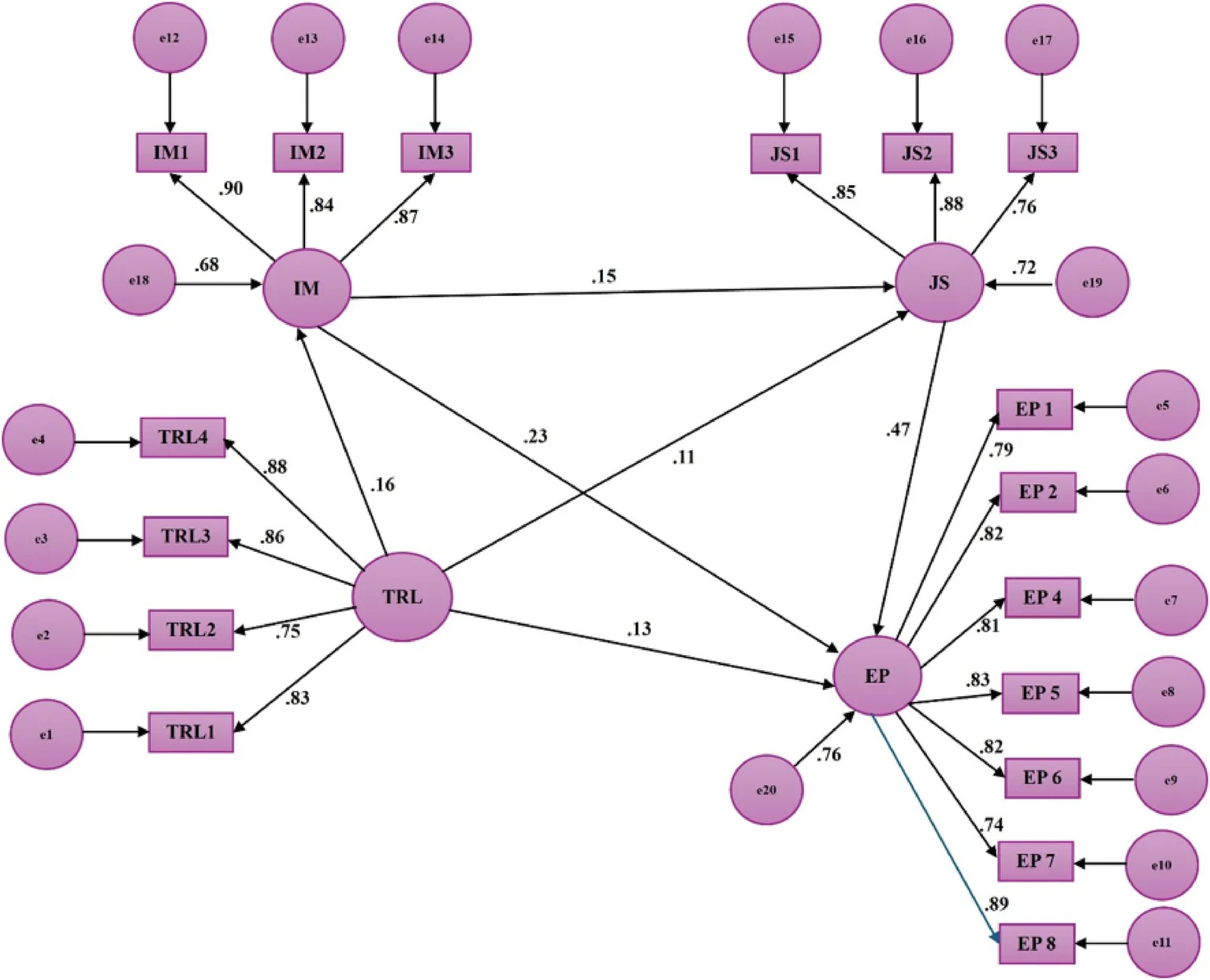
Standardized path coefficients and significance of the model.

**Table 2:** Path coefficients of the standardized model.

Variables	Paths	Variables	Estimates	SE	CR	P
TRL	→	JS	0.106	0.018	5.827	***
TRL	→	EP	0.128	0.019	6.806	***
TRL	→	IM	0.155	0.020	7.949	***
IM	→	JS	0.151	0.022	6.831	***
IM	→	EP	0.232	0.028	8.366	***
JS	→	EP	0.473	0.031	15.397	***
TRL*PC	→	IM	0.428	0.029	14.725	***

Explained variance for each dependent variable (R^2^)						
		IM	JS	EP		
		0.683	0.722	0.764		

Mediating effect of IM between TRL, JS, and EP				
Paths	Estimates	SE	Sobel Z-Values	*p*-values
TRL → IM → JS	0.155 0.151	0.020 0.022	5.138	0.000
TRL → IM → EP	0.155 0.232	0.020 0.028	5.660	0.000
TRL*PC → IM → JS	0.428 0.151	0.029 0.022	6.224	0.000
TRL*PC → IM → EP	0.367 0.232	0.041 0.028	6.080	0.000

TRL = transformational leadership, IM = intrinsic motivation, PC = perceived calling, JS = job satisfaction, EP = employee performance.

This study is geared toward assessing the associations between TRL and JB and EP, and the mediating effect of intrinsic motivation on these relationships among public sector employees in Sierra Leone. Moreover, we tested for the moderating mechanism of the impact of PC on these relationships. The aim is to test whether the effect is greater with a greater PC than with a lesser PC, or vice versa. The suggested research model shows the order of the predicted links. Consequently, we used SEM to “test the relationships (Figure 2; Table 2) by representing TRL as an independent variable and JB and EP as dependent variables. The results show that *TRL* is positively related to *JS* (*β* = 0.106, *p* < 0.01). Hence, H1 is supported. Similarly, *TRL* was positively related to EP among public sector employees (*β* = 0.128, *p* < 0.01). Thus, H2 is supported. The findings also reveal that *TRL* is positively related to *IM*, with the statistical values of (*β* = 0.155, *p* < 0.01) supporting H3.

In the same direct relationships, the results also reveal a positive and statistically significant relationship between *JS* and EP among public sector workers (*β* = 0.473, *p* < 0.01). Therefore, H4 is supported. Furthermore, IM positively correlated with both JS (β = 0.151, *p* < 0.001, H4a supported), and EP (β = 0.232, *p* < 0.001, H4b supported). Finally, JS is positively related to EP (β = 0.473, *p* < 0.001; H6 supported).

Finally, we explored the significance of indirect effects (mediation hypotheses) using the bias-corrected accelerated bootstrap method in SEM, implemented in 5,000 resamples to compute confidence intervals for all indirect pathways (Hayes, 2018; Preacher and Hayes, 2008). An indirect effect is considered significant if its bias-corrected 95% CI does not overlap with 0 (Preacher and Hayes, 2008). This bootstrap-based method is much more powerful and requires far fewer restrictive assumptions than conventional indirect-effect tests (e.g., the Sobel test; MacKinnon et al., 2004). To estimate conditional indirect effects for moderated mediation effects (H7b and H7c), we computed the indirect effects at low, mean, and high levels of the moderator (perceived calling). More specifically, indirect effects were calculated at the low (mean minus one standard deviation), mean, and high (mean plus one standard deviation) levels. We used slope interaction tests to visualize moderation effects (Aiken et al., 1991) across the various levels of calling to examine how the strength of the mediated pathways varied across callings. An indirect effect is moderated if the confidence intervals for each indirect path differ across levels of the moderator.

Using the online Sobel test calculator to compute the mediation effects, “the results show a significant positive mediation effect of *IM* on the relationship between *TRL* and *JS* (*z* = 7.357, *p* < 0.01). Thus, H5 is also supported. Similarly, the results confirm a significant positive mediation effect of *IM* on the relationship between TRL and *EP* (*z* = 5.660, *p* < 0.01). Thus, H6 is also supported”.

Lastly, the high–low analysis of the moderating effects of PC is shown in Figure 3. The slope interaction test was utilized to examine the moderating effect. First, Figure 3 shows the interaction of PC on the relationship between TRL and *IM*. The lines on the graph show that when PC is high among employees, the positive relationship between TRL and IM is considerably strong. Thus, H7a is supported.

**Figure 3:**
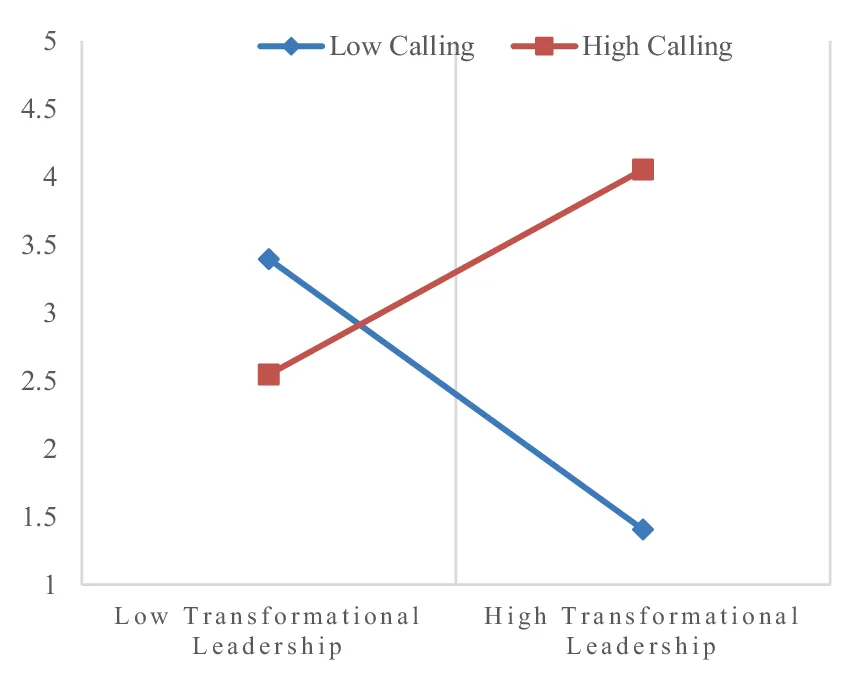
Moderating effects of PC between TRL and IM.

Second, Figure 4 shows that the slope of the relationship between TRL and JS, when mediated by IM, is stronger under high calling than under low calling. Thus, H7b is supported.

**Figure 4:**
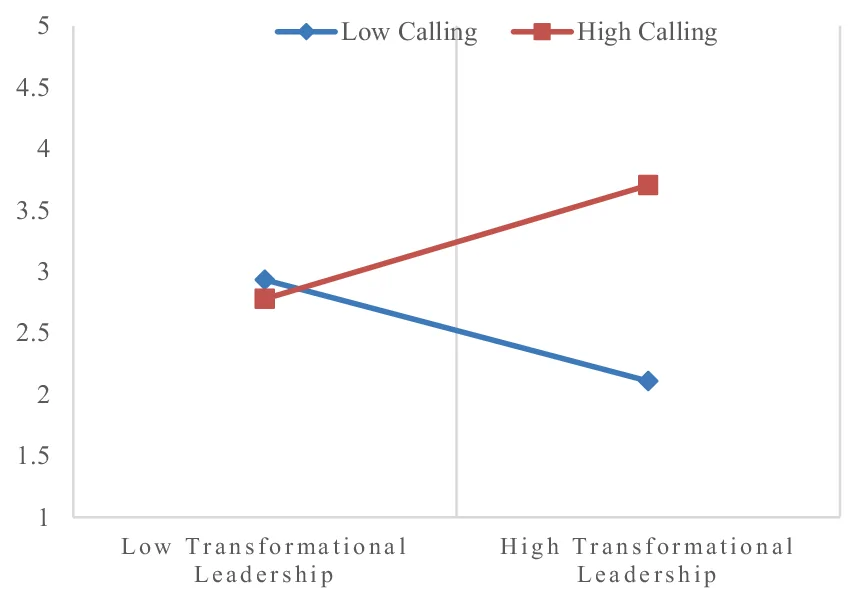
Moderating effects of PC between TRL and JS.

Lastly, Figure 5 indicates that the slope of the relationship between TRL and EP, when mediated by IM, is stronger under high calling than under low calling. Thus, H7c is supported.

**Figure 5:**
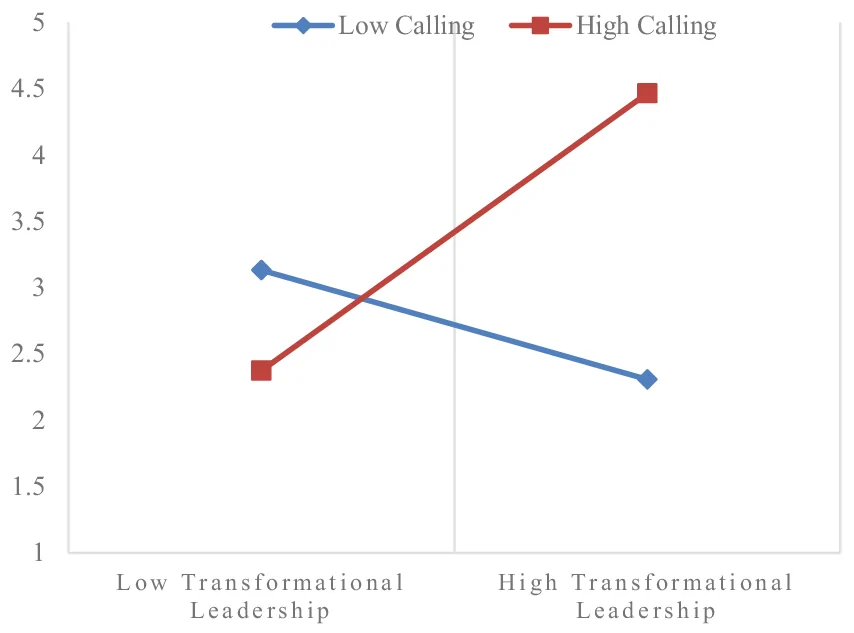
Moderating effects of PC between TRL and EP.

Overall, Table 2 and Figures 3–5 show that the determinants (i.e., TRL and IM) and JS and EP have significant positive effects among public sector employees in Sierra Leone. These results suggest that the research framework and hypotheses of this study are consistent with previous findings.

## 5 Discussion of the results

This study set out to explore the underlying mechanisms and boundary conditions through which TRL impacts JS and EP, focusing on the mediating and moderating roles of IM and PC, respectively. The empirical results confirm all seven hypotheses and demonstrate a theoretically sound and empirically robust moderated mediation model. More importantly, these results further our understanding of effective leadership by identifying the mediating factors that determine when transformational leadership is most powerful and for whom. Instead of promoting transformational leadership as a silver bullet for organizational change, the findings show that it essentially works by tapping into employees’ internal psychological dispositions, namely their motivation to work and a sense of higher purpose.

The findings confirm that TRL is a significant predictor of JS and EP, consistent with previous empirical evidence linking TRL with improved employee attitudes and behaviors (Bass and Riggio, 2006; Buil et al., 2018). Transformational leaders enhance employee empowerment, build trust, and foster emotional commitment, thereby translating into positive work attitudes and enhanced performance outcomes (Park and Park, 2019; Mohammad et al., 2011). In this study, Sierra Leonean public sector employees who reported greater exposure to TRL behaviors also exhibited higher levels of satisfaction and productivity, underscoring the applicability of TRL even in highly bureaucratic, resource-constrained settings.

A particularly noteworthy finding is the significant mediating role of IM in the relationship between TRL and JS and EP. The theoretical significance of these findings lies in their validation of self-determination theory (SDT) as an explanatory mechanism for transformational leadership’s effects. This finding aligns with SDT, which posits that individuals are most motivated and productive when their psychological needs for autonomy, competence, and relatedness are fulfilled (Deci and Ryan, 2000). Transformational leaders satisfy these needs by articulating a shared vision, recognizing individual contributions, and providing opportunities for professional growth (Nguyen T. P. L. et al., 2022; Nguyen T. T. et al., 2022; Mahmood M. et al., 2019). Prior studies have shown that such leadership behaviors stimulate employees’ internal drive, thereby fostering a sense of ownership and meaning in their work (Gagné et al., 2022; Koh et al., 2019). Our findings affirm that when employees feel intrinsically motivated, when they work not for rewards but for internal satisfaction, they are more likely to experience higher levels of job satisfaction and demonstrate superior performance. In line with foundational work conducted by Deci and Ryan (2000), the findings showed that transformational leaders increase job satisfaction and performance not through controlling behaviors or extrinsic incentive structures but rather through supportive social contexts that meet basic employee needs for autonomy, competence, and relatedness. In the context of relatively centralized decision-making, resource constraints, and limited formal opportunities for advancement in public-sector jobs in Sierra Leone. This discovery holds specific theoretical significance. It indicates that even in bureaucracies/organizations with an unquestioned hierarchy, you can spark intrinsic motivation by treating workers as human beings, not just as subjects of your formal power or material benefits. This is a significant extension of SDT that has been tested extensively in Western contexts but has only nascent applications in African public institutions, where constraints on extrinsic incentives are structural rather than theoretical.

Another critical contribution of this study is the introduction of PC as a moderating variable that influences TRL effectiveness. This idea aligns with existing literature, suggesting that leadership effectiveness is not uniform across individuals and that contextual or psychological factors, such as calling, can shape how leadership messages are received (Duffy et al., 2018). In particular, the results demonstrate that the positive influence of TRL on IM is significantly stronger among employees with a higher sense of calling. This finding supports Fredrickson's (2001) broaden-and-build theory, which posits that individuals with high levels of positive emotion (in this case, calling) are likely to broaden their thought–action repertoires and build personal resources. The results show that the positive indirect effect of transformational leadership on intrinsic motivation is higher for employees who perceive work as a calling than for employees with low calling. This is more than a statistical interaction; it is a principle that emerges from the way employees are already oriented toward purpose before encountering any leadership influence. Based on Fredrickson’s theory, it can be concluded that these individuals with a high degree of perceived calling have more expansive emotional and cognitive resources, allowing our participants to reconstruct messages from transformational leaders as meaningful and purpose-aligned. They are conditioned to discover the connections between the leader’s articulated vision and their inner instinct for purpose, thereby increasing its motivational potential. On the contrary, employees with low perceived calling might perceive the same leadership behaviors as irrelevant (push) to their intrinsic mission and response. These employees are more emotionally and cognitively open to the messages of transformational leaders, making them more responsive and motivated (Greimel et al., 2023; Wrzesniewski et al., 1997). Conversely, those with low PC may be less receptive to leadership influence, thereby diminishing the intended outcomes of TRL.

The moderated mediation model supported in this study contributes a markedly complex understanding of how leadership, motivation, and individual purpose interact. Employees with a strong sense of calling appear to effectively internalize the mission and vision communicated by transformational leaders, thereby enhancing their intrinsic motivation and leading to substantial job satisfaction and performance. This result is consistent with Desmidt and Prinzie (2018), who found that transformational leaders can amplify employees’ sense of mission valence through symbolic behavior and visionary communication. Similarly, Jacobsen et al. (2013) noted that changes in how employees perceive their roles driven by social cues from leadership can be more influential than objective task characteristics. In public organizations, where jobs may be rigid and change-resistant, leveraging calling as an emotional and psychological resource could be essential for driving performance improvements.

These findings have significant contextual implications for Sierra Leone and other public sectors in similarly situated developing economies. Public sector organizations in resource-constrained environments rarely have the resources to provide salaries competitive with the private sector, and they often fall short of providing an extensive set of material incentives, traditional “carrots” that many private sector organizations count on to drive performance. The findings of this research indicate that this type of resource constraint is not fatal to organizational effectiveness. Public sector leaders in Sierra Leone should leverage transformational behaviors, including articulating visions, providing intellectual stimulation, and giving individualized attention, to trigger employees’ intrinsic motivation to perform their roles, especially among those who see their public service as a calling that resonates with their personal values. This is NOT an argument against structural and substantive reforms, which are still necessary. Instead, it is a data-driven claim that, at the current resource-constrained level, leadership quality and psychological empowerment are strong performance drivers, at least from the perspective that they entail no material cost. The fact that the indirect effect of TRL on EP through IM is both substantial and significant means this motivational pathway is not only conceivable in theory but also established in practice. This insight has immediate policy relevance for Sierra Leone’s Ministry of Finance, the Ministry of Health, and other service-delivery organizations: investments in transformational leadership development may yield employee engagement and public service quality dividends comparable to income gains on a relative scale within tight budgets.

This study also clarifies and, in some respects, qualifies prior conclusions drawn from transformational leadership research. Previous meta-analyses and empirical research on transformational leadership (Bass and Riggio, 2006; Judge and Piccolo, 2004) showed a fairly consistent predictive relationship with positive employee outcomes, but the early studies modeled this relationship as if it were invariant across contexts and individuals. However, the moderated mediation framework of the current study demonstrates that this conclusion is incomplete. The conditional indirect effects across levels of perceived calling in this study explain the large variability seen across workplace environments for the practical significance of transformational leadership. The effect heterogeneity of transformational leadership is underrepresented in the contemporary literature (Uhl-Bien et al., 2007), and this gap is explicitly addressed by this study, which shows that calling is an important contextual moderator that distinguishes effective from less effective transformational leadership across all three studies.

This study deepens our understanding of leadership effectiveness in the public sector by highlighting the importance of psychological mechanisms and boundary conditions. The findings show that transformational leaders can significantly enhance job outcomes by nurturing employees’ IM and reinforcing a sense of calling. These findings provide empirical support for leadership models that go beyond transactional exchanges and focus on meaning-making, vision-sharing, and individual growth (Shamir et al., 1993; Su et al., 2020). They also respond to calls for additional context-sensitive leadership theories that reflect individual variability and emotional resonance (Uhl-Bien et al., 2007; Eliyana et al., 2019). In contexts such as Sierra Leone, where limited resources and bureaucratic rigidity often constrain public institutions, the ability of leaders to inspire, motivate, and connect with their followers’ deep values could be key to transforming organizational culture and outcomes.

### 5.1 Theoretical contributions

This study contributes significantly to the leadership and organizational behavior literature by offering a context-specific understanding of how TRL operates through psychological mechanisms, specifically IM, to influence JS and EP. Although previous research has established the direct effects of TRL on employee outcomes (Bass and Riggio, 2006; Mohammad et al., 2011; Park and Park, 2019), the current study advances the theory by validating a mediated pathway, grounded in SDT (Deci and Ryan, 2000), in which IM serves as a key conduit for TRL’s effects. This mediational role reinforces the view that leadership effectiveness is not solely a result of directive behaviors but of a leader’s ability to activate internal psychological states (Gagné et al., 2022; Nguyen T. P. L. et al., 2022; Nguyen T. T. et al., 2022). By focusing on internal, self-directed motivation rather than external incentives, this study helps reframe TRL’s role as a psychological catalyst in organizational settings, particularly in resource-constrained public institutions.

Furthermore, this research extends SDT by integrating PC as a moderating variable, offering a moderated mediation model that reveals when and for whom TRL is most effective. Although SDT emphasizes the universal importance of autonomy, competence, and relatedness in fostering motivation, this study demonstrates that individual differences in PC shape how employees interpret and respond to TRL behaviors (Duffy et al., 2018; Fredrickson, 2001). This finding highlights a key boundary condition: TRL may fail to produce motivational or performance outcomes in individuals with low levels of calling, even when other psychological needs are met. As such, this research supports a markedly contingent leadership model, aligning with the growing body of research suggesting that leadership effectiveness depends on both behaviors and followers’ internal orientations and emotional states (Wrzesniewski et al., 1997).

Lastly, this study makes a contextual theoretical contribution by validating the preceding relationships within the public sector of a developing country, a context often overlooked in leadership research. The vast majority of TRL studies have been conducted in Western or corporate environments, where employee autonomy and leader-member exchange may differ structurally (Boer et al., 2016; Wong and Berntzen, 2019). This study, by focusing on public institutions in Sierra Leone, addresses a geographical and institutional gap in the literature and shows that TRL remains effective even in bureaucratic, hierarchical settings when supported by intrinsic motivation and a strong sense of calling. This finding suggests that universal leadership theories, such as TRL and SDT, must be applied with consideration of cultural, institutional, and individual differences, thereby pushing the boundaries of theory toward globally inclusive models of leadership.

### 5.2 Practical/managerial contributions

The findings of this study have several practical implications for public sector leadership and human resource management, particularly in developing-country contexts such as Sierra Leone. First, the evidence that TRL significantly enhances JS and EP through IM highlights the urgent need for public institutions to invest in leadership development programs. Leaders should be trained in technical or administrative competence and also in transformational behaviors, such as idealized influence, inspirational motivation, individualized consideration, and intellectual stimulation (Bass and Riggio, 2006). By nurturing these competencies, public sector organizations can empower their leaders to inspire purpose, autonomy, and commitment among employees—critical levers for enhancing performance in bureaucratic and resource-limited settings.

Second, the current study underscores the importance of recognizing and fostering employees’ sense of calling to enhance the effectiveness of TRL. Given that calling was found to moderate the relationship between TRL and IM, organizations should develop systems to identify employees’ personal values, career goals, and intrinsic drivers during recruitment and performance appraisal processes. HR managers could incorporate tools, such as career coaching, reflective assessments, and values-alignment workshops, to help employees articulate and connect their sense of purpose to organizational missions. Doing so would improve intrinsic motivation and also create a markedly resilient, engaged, and committed workforce capable of sustaining performance under difficult circumstances.

Lastly, this research provides actionable insights for policymakers and top-level administrators in the public sector who often seek to improve service delivery without significant structural reforms or budget increases. By focusing on non-monetary levers of motivation, such as TRL behaviors and fostering a sense of calling, leaders can achieve measurable improvements in employee outcomes without relying solely on financial incentives. This aspect is particularly relevant in the African public sector, where budgetary constraints limit extrinsic rewards. In such environments, psychological empowerment and value-based leadership emerge as powerful, cost-effective strategies for improving institutional effectiveness. Therefore, integrating TRL principles into public management practices, talent development, and succession planning can lead to sustained improvements in job satisfaction and performance across the sector.

### 5.3 Limitations and future research

Although this study makes an important contribution to the theory of leadership and to practical implications for public sector practice, caution should be taken in interpreting the findings, as it has some limitations. A few limitations need to be noted regarding this study: the cross-sectional, single-source survey design does not permit robust causal inference. While the model with leadership as a predictor, intrinsic motivation as a mediator, and outcomes as consequences is theoretically logical, our current design does not rule out definite reverse causal pathways or unmeasured confounding. For example, one might argue that employees who feel more satisfied with their jobs in retrospect assess their leaders as more transformational than they actually are. Future longitudinal designs that use lagged measures or experimental manipulations of leadership framing would greatly enhance causal inference (Spector, 2019). Third, despite procedural and statistical remedies to the potential CMV concern detailed in Sections 3.3 and 4.1, single-source self-report measurement may still suffer from common method variance (CMV). Additional researchers should remain cognizant of this (Podsakoff et al., 2003). While procedural remedies were employed, mandated respondent anonymity, separating predictor and criterion items by a few pages, and using validated multi-item scales (Podsakoff et al., 2003), self-report measures of leadership perception, motivation, and performance satisfaction are still subjective variables that can be influenced by social desirability bias or implicit theories about how good leadership should look. This issue would have been considerably ameliorated in future studies if supervisor or peer ratings of leadership and performance, or objective organizational metrics (e.g., absenteeism, turnover, productivity indices), had been included (Conway and Lance, 2010). Third, the generalizability of our findings is limited by context and time. Because data collection was limited to six public ministries in Freetown, Sierra Leone, results may not generalize to the private sector or to resource-constrained rural public organizations, nor be representative of other international contexts with different institutional and cultural features (Karimi et al., 2023; Wong and Berntzen, 2019). Additionally, our cross-sectional design limits us to a snapshot in time of an extremely fluid public administration environment. Because policy changes, leadership transitions, and resource fluctuations constantly alter public sectors, these findings cannot explain long-term temporal trends or behavioral responses to organizational crises. Fourth, this study assumes that the relationships between the measures and the tested theory are equally meaningful across all demographic and occupational subsets within the sampled ministries. However, the effectiveness of transformational leadership, the salience of intrinsic motivation, and even the relevance of calling may vary significantly across occupational levels (e.g., senior administrators vs. frontline service providers), with gender, educational attainment, and tenure all potentially making a difference. Further multigroup structural equation modeling of these conditional effects would yield more detailed information about which employee groups are better targets for transformational leadership strategies (Kline, 2015). Fifth, though the moderated mediation model examined in this study has strong theoretical grounding and empirical support, it is merely one potential pathway and boundary condition through which transformational leadership impacts outcomes. Other possibly critical (Eliyana et al., 2019; Khan and Khan, 2019) mediators (e.g., organizational identification and trust in leadership; psychological empowerment beyond intrinsic motivation) or moderators (e.g., organizational justice, transformational change context, team cohesion) that may help explain leadership effects were not included in the model. Lastly, although this study employs structural equation modeling (SEM) to investigate the symmetrical net effects of transformational leadership (TRL) on job satisfaction (JS) and employee performance (EP), future research might use fuzzy-set Qualitative Comparative Analysis (fsQCA). The configurational perspective acknowledges asymmetric, equifinal pathways to organizational performance above the desired level. In particular, fsQCA may demonstrate how synergistic combinations of leadership behaviors, internal motivation (IM), and perceived calling (PC) combine to determine performance and satisfaction attributes, even where structural or psychological deficiencies within the civil service workforce create barriers to the achievement of beneficial outcomes (Kallmuenzer et al., 2025). Despite these limitations, the current study makes important contributions through: (a) testing a new moderated mediation model in African public sector contexts, (b) providing empirical evidence that perceived calling is an important moderator of leader effects, and (c) showing that transformational leadership influences employees intrinsic motivation and work outcomes even under severely resource constrained institutional settings with some specific guidance for leadership development practices within West African public sectors.

## 6 Conclusion

This study provides robust empirical evidence that TRL significantly enhances JS and EP, primarily by fostering IM among employees. The moderating role of calling further refines our understanding, showing that employees with a strong sense of purpose respond more favorably to TRL, resulting in greater motivation and better outcomes. These findings are particularly relevant for public organizations in developing-country contexts, where leadership quality and employee engagement are critical yet underexamined factors. By integrating SDT with constructs of leadership and calling, this study builds a comprehensive framework that deepens our understanding of the psychological and contextual factors driving employee outcomes. In principle, the research moves leadership scholarship away from straightforward main-effect models to contingent models that specify when and for whom leadership effects are strongest, directly addressing recent calls for more complex, psychometric-informed leadership theories (Uhl-Bien et al., 2007). In practical terms, what they show is that in resource-constrained environments, public sector leaders can create significant improvements in engagement and performance by engaging in transformational behaviors that appeal to employees’ sense of purpose and intrinsic motivation. This also provides a strong, inexpensive incentive for better performance in Sierra Leone and in at least similar developing economies. Future research could expand on this model by (1) using longitudinal designs with several waves of measurement to reinforce causal inferences, (2) including multi-source data (supervisor and peer ratings of leadership and performance) to reduce common method bias, (3) testing the model across occupational levels, genders, and tenure groups to investigate moderated mediation; and (4) examining mediators other than need for meaning or moderators over potential moderators such as organizational justice pervade as well as change context further explains effectiveness. Overall, this study advances academic theory and also provides actionable guidance for public sector leaders seeking to build markedly engaged, satisfied, and high-performing workforces.

## Data Availability

The raw data supporting the conclusions of this article will be made available by the authors, without undue reservation.
